# Hemolysis and Its Clinical Implications in Septic Patients with Acute Respiratory Failure

**DOI:** 10.3390/jcm14103493

**Published:** 2025-05-16

**Authors:** Wojciech Bąkowski, Jakub Śmiechowicz, Anna Lemańska-Perek, Barbara Dragan, Waldemar Goździk, Barbara Adamik

**Affiliations:** 1 Clinical Department of Anesthesiology and Intensive Therapy, Wroclaw Medical University, Borowska 213, 50-556 Wroclaw, Poland; jakub.smiechowicz@umw.edu.pl (J.Ś.); barbara.dragan@umw.edu.pl (B.D.); waldemar.gozdzik@umw.edu.pl (W.G.); 2 Department of Chemistry and Immunochemistry, Wroclaw Medical University, M. Sklodowskiej-Curie 48/50, 50-369 Wroclaw, Poland; anna.lemanska-perek@umw.edu.pl

**Keywords:** hemolysis, sepsis, septic shock, ARDS, ECMO, CRRT

## Abstract

**Background**: Hemolysis during sepsis may be driven by patient-specific factors, including the intensity of the inflammatory response and the etiology of infection, as well as treatment-related factors, such as the use of extracorporeal life-support devices. **Methods**: We evaluated the incidence of hemolysis—reflected by decreased plasma levels of haptoglobin and hemopexin—in a cohort of septic patients with acute respiratory failure (*n* = 50) admitted to the intensive care unit (ICU). **Results**: Hemolysis was observed in 60% of patients. Its incidence was significantly higher among those with septic shock (86%) and those receiving extracorporeal membrane oxygenation (ECMO) therapy (81%). While continuous renal replacement therapy (CRRT) alone did not increase the incidence of hemolysis, its combination with ECMO was associated with hemolysis in 100% of those treated. Logistic regression analysis identified low haptoglobin levels (odds ratio [OR] 27.1), advanced age (OR 1.2), and stage 3 acute kidney injury (OR 22.2) as significant predictors of mortality. **Conclusions**: These findings highlight the clinical relevance of monitoring hemolysis in septic patients. Given the routine availability of haptoglobin and hemopexin assays in most hospital laboratories, these biomarkers offer practical and accessible tools for the detection and monitoring of hemolysis in critically ill patients.

## 1. Introduction

Hemolysis during sepsis may be induced both by patient-specific factors related to the intensity of the inflammatory response or the etiology of infection, as well as by factors related to therapy, such as the use of extracorporeal life-support devices. To mitigate the harmful effects of hemolysis, the body employs scavenger proteins, including haptoglobin and hemopexin, which serve as protective mechanisms and potential biomarkers of hemolysis. Haptoglobin binds cell-free hemoglobin, forming a large-molecular-size complex that interacts with the CD163 receptor on macrophages and monocytes, facilitating endocytosis and degradation in the liver and spleen [1]. Simultaneously, heme released from cell-free hemoglobin is sequestered by hemopexin, and then degraded by hepatocytes. However, in severe hemolysis, these clearance mechanisms can become saturated, leading to scavenger protein depletion and the accumulation of cell-free hemoglobin and heme in the intravascular compartment. Additionally, hemoglobin dimers formed during hemolysis may translocate into the extravascular space. Consequently, the harmful reactivity of cell-free hemoglobin and heme extends beyond the intravascular compartment, and may lead to severe physiological consequences that exacerbate sepsis-related organ dysfunction [2].

Cell-free hemoglobin exerts harmful effects through multiple pathogenic mechanisms. It promotes oxidative stress by generating free radicals that damage lipids, proteins, and DNA, thereby exacerbating inflammation [3]. These free radicals also compromise red blood cell (RBC) membranes, increasing their fragility and susceptibility to hemolysis. Additionally, cell-free hemoglobin binds nitric oxide, reducing its bioavailability and leading to vasoconstriction, elevated vascular resistance, impaired microcirculation, and worsening tissue hypoxia. Moreover, heme acts as an activator of Toll-like receptor 4 and the NF-κB signaling pathway, further amplifying the inflammatory response in sepsis [4].

Hemolysis can exacerbate inflammation and contribute to multiorgan failure, potentially worsening clinical outcomes in septic patients [5,6]. Although elevated levels of cell-free hemoglobin are associated with poor prognosis in sepsis, intravascular hemolysis is rarely monitored in routine ICU practice. Rather than directly measuring cell-free hemoglobin, we assessed haptoglobin and hemopexin, indirect yet clinically accessible markers of hemolysis, since their measurement is routinely performed in our hospital laboratory. Haptoglobin depletion indicates intravascular hemolysis, as it binds and clears excess free hemoglobin, while reduced hemopexin levels suggest prolonged hemolysis, reflecting its function in scavenging free heme. Monitoring these markers may offer valuable clinical insight into the severity of hemolysis and its potential impact on patient outcomes. Previous studies have demonstrated a relationship between haptoglobin and hemopexin concentrations and clinical outcomes in patients with sepsis. In a study by Janz et al., reduced levels of both haptoglobin and hemopexin were significantly associated with increased hospital mortality among patients with detectable plasma cell-free hemoglobin [7]. Verheij et al. observed significantly lower hemopexin levels and higher haptoglobin levels in septic patients upon ICU admission compared to healthy volunteers; however, neither protein was predictive of sepsis severity [8]. More recently, in a study involving ICU patients undergoing veno-venous extracorporeal membrane oxygenation (ECMO), haptoglobin levels were associated with vascular tone modulation, ventilator-associated pneumonia (VAP), and ICU mortality [9]. Specifically, lower haptoglobin levels were linked to increased pulmonary and systemic vascular resistance, a higher incidence of VAP, and greater ICU mortality. In this study, we investigated the incidence of hemolysis, reflected by decreased haptoglobin and hemopexin levels, in septic patients with acute respiratory failure admitted to the ICU. Specifically, we examined hemolysis in relation to potential contributing factors, including septic shock, infection with hemolytic bacteria, and treatment with extracorporeal life-support devices. Given the previously reported association between hemolysis and adverse clinical outcomes, we also aimed to develop a predictive model for mortality in the analyzed cohort.

## 2. Materials and Methods

### 2.1. Patients

This retrospective observational study was conducted in an intensive care unit of Wroclaw University Hospital between January 2021 and December 2022 (spanning 24 months). The study protocol received approval from the Bioethics Committee of Wroclaw Medical University (Approval No. KB–996/2021; date: 13 December 2021), and the research adhered to the principles outlined in the 1975 Declaration of Helsinki, as revised in 2013. Informed consent was waived by the Bioethics Committee due to the retrospective nature of this study. All data were extracted from hospital records. Consecutive adult patients (≥18 years) admitted with sepsis and acute respiratory failure were screened for eligibility. Sepsis was defined, according to Sepsis-3 criteria, as suspected or documented infection and acute organ dysfunction with an increase in SOFA score of ≥2 points [10]. Acute respiratory failure was diagnosed based on a PaO_2_/FiO_2_ ratio <300 mmHg or clinical signs of respiratory distress requiring invasive mechanical ventilation. Inclusion criteria were as follows: (I) age ≥18 years; (II) diagnosis of sepsis at the time of ICU admission; (III) acute respiratory failure requiring mechanical ventilation at the time of ICU admission; and (IV) availability of plasma haptoglobin and hemopexin measurements initiated within the first 24 h of ICU admission. Exclusion criteria included pre-existing hematological disorders, renal or hepatic failure, and trauma.

### 2.2. Data Collection

Baseline data were prospectively collected, including demographics (age, sex), comorbidities (hypertension, diabetes, coronary artery disease, obesity, malignancy), the severity of illness (APACHE II and SOFA scores), the source and type of infection (viral, bacterial, or mixed), the presence of septic shock, and the need for extracorporeal membrane oxygenation (ECMO) or continuous renal replacement therapy (CRRT). To calculate the APACHE II score for each patient, laboratory parameters—such as blood pH, serum sodium, potassium, creatinine, hematocrit, white blood cells, oxygen partial pressure, and inhaled oxygen fraction—and values of clinical parameters—such as the Glasgow coma scale, heart rate, respiratory rate, body temperature, and mean arterial pressure—were used. Organ failure was assessed using the Sequential Organ Failure Assessment (SOFA) score, which evaluates six organ systems: cardiovascular (mean arterial pressure and vasopressor requirements), respiratory (PaO_2_/FiO_2_ ratio), hepatic (serum bilirubin concentration), renal (serum creatinine concentration), neurological (Glasgow Coma Scale), and coagulation (platelet count). Microbiological samples were obtained from blood, respiratory specimens, and other indicated sites. Viral pathogens were identified using real-time PCR assays targeting SARS-CoV-2 and influenza A virus.

### 2.3. Hemolysis Measurements

Blood samples for haptoglobin and hemopexin were collected within 24 h of ICU admission and monitored daily for the next seven days in the hospital laboratory. Plasma haptoglobin and hemopexin concentrations were measured using commercial immunonephelometric assays with N Antiserum to Human Haptoglobin and N Antiserum to Human Hemopexin, respectively (Atellica^®^ NEPH 630 System, Siemens Healthineers AG, Forchheim, Germany) [11,12]. Both assays are CE IVD-certified and are routinely used in our hospital laboratory for detecting hemolysis in patient samples. The reference ranges are 0.30–2.00 g/L for haptoglobin and 0.50–1.15 g/L for hemopexin. Hemolysis was defined as a haptoglobin and/or hemopexin concentration below the reference range. Other routine laboratory tests, including complete blood count, C-reactive protein (CRP), procalcitonin (PCT), fibrinogen, D-dimer, lactate dehydrogenase (LDH), and antithrombin III (AT III) activity, were also recorded. Results of the peripheral blood smears were extracted from hospital records and examined for the presence of schistocytes.

### 2.4. Patient Management

All patients were treated in accordance with Surviving Sepsis Campaign guidelines, including the use of vasopressors, fluid therapy, steroids, antibiotics, and continuous renal replacement therapy [13]. Sepsis and septic shock were diagnosed based on Sepsis-3 criteria [10]. Acute respiratory distress syndrome (ARDS) was determined according to the Berlin ARDS [14]. ECMO was initiated for patients with refractory hypoxemia (PaO_2_/FiO_2_ < 100 mmHg) after multidisciplinary team consultation. ECMO cannulation was performed percutaneously. The V-V ECMO circuit included a Quadrox-i adult microporous membrane oxygenator (MAQUET Holding BV & Co., KG, Rastatt, Germany), and either a CardioHelp or continuous life-support set with a Rotaflow II base unit (MAQUET Holding BV & Co., KG, Rastatt, Germany). Verification of proper cannula positioning was performed via a chest *X*-ray, ultrasound examination, and clinical assessment of the patient’s condition. The general goal of ECMO therapy was to prevent hypoxemia and hypercapnia; to maintain an oxygen saturation above 88%, normoxemia, and normocapnia in the arterial blood gas; and to prevent acid–base disorders. Continuous renal replacement therapy (CRRT) was initiated according to the KDIGO criteria for stage 3 acute kidney injury [15].

### 2.5. Statistical Analysis

Continuous variables were presented as medians with interquartile ranges (IQRs, 25th and 75th percentiles), and categorical variables as counts and percentages. The distribution of the variables was not normal based on a Shapiro–Wilk test. Comparisons between groups were performed using the Mann–Whitney U test for continuous variables, and using contingency tables with chi-square or Fisher’s exact tests for categorical variables. Trends in haptoglobin and hemopexin concentrations over the observation period were analyzed using repeated-measures ANOVA. Univariate and multivariate logistic regression analyses identified factors associated with ICU mortality. Variables with *p* < 0.05 were included in the multivariate logistic regression models. The results were expressed as odds ratios (OR) with 95% confidence intervals (CI). A two-sided *p*-value < 0.05 was considered statistically significant. Statistical analyses were performed using Statistica v.13.3 (TIBCO Software Inc., Palo Alto, CA, USA), under a license held by Wroclaw Medical University.

## 3. Results

**Figure 1:**
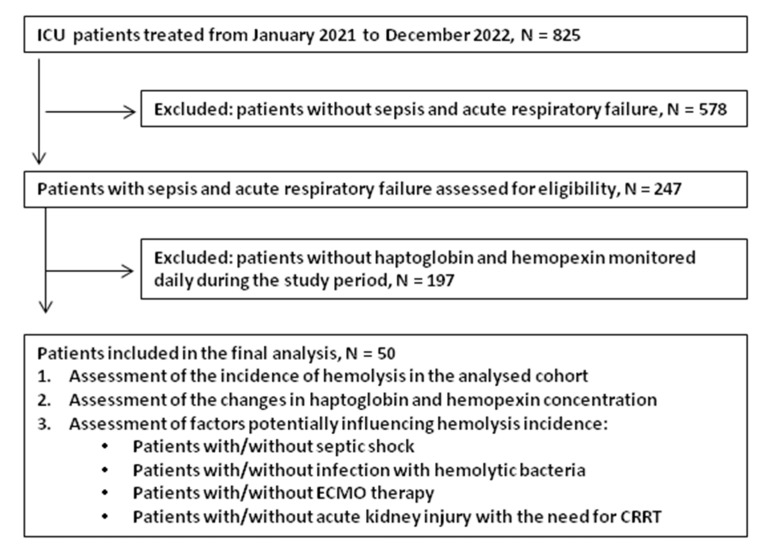
Flow diagram of study.

Between January 2021 and December 2022, 247 septic patients admitted with acute respiratory failure were assessed for eligibility based on the inclusion and exclusion criteria. Of these, only 20% had haptoglobin and hemopexin checked on ICU admission and monitored daily thereafter, and these patients were included in the final analysis. Figure 1 is a flow diagram of the study participants.

The median patient age was 47 years (IQR 38–59), and 66% of the patients were male. On ICU admission, all patients presented with acute respiratory failure, and ARDS was confirmed in 90%. Sepsis on ICU admission was diagnosed in all patients, and septic shock was diagnosed in 58% based on the Sepsis-3 criteria [guideline]. A viral infection upon ICU admission was confirmed in 46 patients, including SARS-CoV-2 and influenza virus type A. Bacterial pathogens upon ICU admission were identified in 20 patients, with 16 results involving mixed bacterial–viral infections, and 4 involving bacterial infections only. Peripheral blood smear results, obtained from hospital records and evaluated for the presence of schistocytes, showed that smears were performed in 26% of patients (13 out of 50), and no schistocytes were identified in any of the examined samples. Patients were stratified into two study groups based on the presence of hemolysis, defined as a plasma haptoglobin or hemopexin concentration below the reference range at any study time point. The reference ranges provided by hospital laboratory were 0.30–2.00 g/L for haptoglobin and 0.50–1.15 g/L for hemopexin. Group 1 comprised patients with hemolysis (*n* = 30), whereas Group 2 included those without hemolysis (*n* = 20). Baseline characteristics of the study population are summarized in Table 1.

### 3.1. Haptoglobin and Hemopexin in the Analyzed Cohort

**Figure 2:**
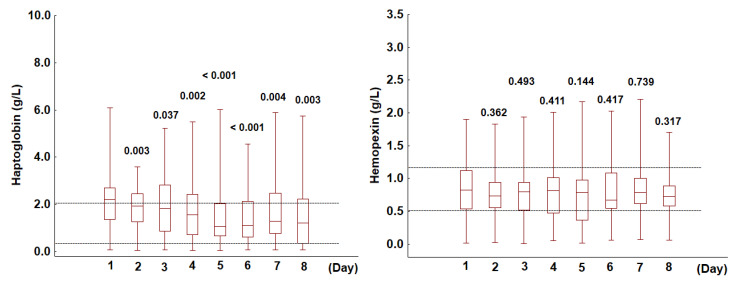
Changes in haptoglobin and hemopexin levels in the analyzed cohort over the eight-day observation period. The dotted lines represent the upper and lower reference ranges for haptoglobin (0.30–2.00 g/L) and hemopexin (0.5–1.15 g/L). The box plots depict the median values (midline) and interquartile ranges (box) between the 25th and 75th percentiles, while the whiskers indicate the minimum and maximum values. For both parameters, the *p*-values shown on days 2–8 refer to comparisons with baseline values measured on admission to the ICU.

As haptoglobin binds extracellular hemoglobin (Hb), which serves as a source of heme subsequently bound by hemopexin, deficiencies in these proteins are indicative of hemolysis. To detect hemolysis, the concentrations of both markers were measured over an eight-day observation period. On admission to the ICU, haptoglobin levels were outside the reference range in the majority of patients (64%), with 52% exhibiting elevated levels and 12% displaying levels below the reference range. Only 36% of patients had haptoglobin concentrations within the normal range, i.e., from 0.30 to 2.00 g/L. Notably, upon ICU admission, unlike haptoglobin, hemopexin concentrations were within the reference range, i.e., from 0.50 to 1.15 g/L, in most patients (62%), below the reference range in 20% of patients, and above the reference range in 18%. The temporal dynamics of haptoglobin and hemopexin concentrations varied throughout the study period. Compared to the baseline values measured upon ICU admission, haptoglobin levels declined significantly over the subsequent days (*p* < 0.001). In contrast, hemopexin concentrations remained relatively stable (*p* = 0.772), with no statistically significant variations observed throughout the observation period (Figure 2). In the following paragraphs, we examine potential factors contributing to haptoglobin or hemopexin deficiency during the eight-day ICU treatment.

### 3.2. Evaluation of Factors Potentially Influencing Hemolysis Incidence

**Figure 3:**
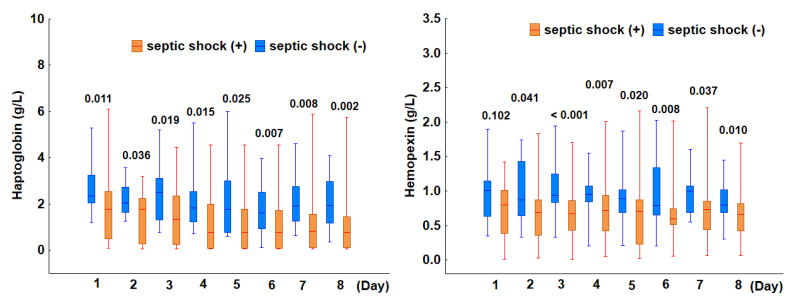
Comparison of haptoglobin (**left panel**) and hemopexin (**right panel**) levels between patients with and without septic shock. The box plots depict the median values (midline) and interquartile range (box) between the 25th and 75th percentiles, while the whiskers indicate the minimum and maximum values. For both parameters, the *p*-values shown for days 1–8 represent comparisons between patients with and without septic shock.

**Figure 4:**
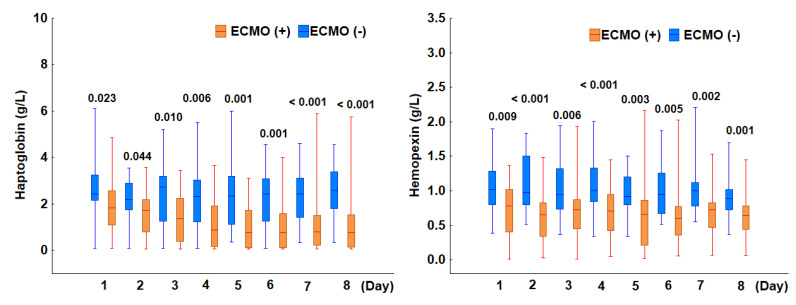
Comparison of haptoglobin (**left panel**) and hemopexin (**right panel**) levels between patients with and without ECMO. The box plots depict the median values (midline) and interquartile range (box) between the 25th and 75th percentiles, while the whiskers indicate the minimum and maximum values. For both parameters, the *p*-values shown for days 1–8 represent comparisons between patients with and without ECMO.

We assessed the incidence of hemolysis in relation to various potential contributing factors, including septic shock, infection with hemolytic bacteria, ECMO therapy, and acute kidney injury with the need for CRRT. Additionally, we compared changes in the concentrations of two hemolysis markers, haptoglobin and hemopexin, based on the presence or absence of the aforementioned factors (Figure 2, Figure 3 and Figure 4).

#### 3.2.1. Incidence of Hemolysis in Patients with Septic Shock

To investigate the association between plasma haptoglobin and hemopexin levels and septic shock, the incidence of hemolysis and the levels of both markers were compared in patients with and without shock. Upon ICU admission, 29 patients (58%) met the Sepsis-3 criteria for septic shock. The incidence of hemolysis was significantly increased in patients with septic shock. Specifically, 86% (25 of 29) of patients with shock exhibited hemolysis, compared to only 24% (5 of 21) of those without shock (*p* < 0.001). Moreover, both indicators of hemolysis, haptoglobin and hemopexin, remained consistently lower in patients with septic shock than in those without shock throughout the eight-day observation period (Figure 3). These findings suggest that septic shock at ICU admission is strongly associated with an increased incidence of hemolysis.

#### 3.2.2. Incidence of Hemolysis in Patients with Bacterial Infections

Hemolysins, toxins produced by certain bacteria, may contribute to intravascular hemolysis. In our cohort, hemolysin-producing bacteria were identified in six patients (12%). However, there was no significant difference in the incidence of hemolysis between patients with hemolytic bacterial infections and those without (*p* = 0.544). Furthermore, haptoglobin and hemopexin levels remained comparable between the two groups over the eight-day observation period.

#### 3.2.3. Incidence of Hemolysis in Patients Treated with ECMO

ECMO, as an extracorporeal life-support device, has the potential to induce hemolysis. To investigate this, we analyzed haptoglobin and hemopexin levels in ICU patients with and without ECMO initiated at admission. All patients were diagnosed with acute respiratory failure upon ICU admission and required mechanical ventilation for lung support. The most severe respiratory failure, indicated by the lowest PaO_2_/FiO_2_ ratio (<100), was recorded in 74% of patients (*n* = 37), whereas a PaO_2_/FiO_2_ ratio within the range of 100–199 was observed in 26% (*n* = 13). A total of 32 patients received ECMO, initiated within 24 h of ICU admission. Among them, 25 had a baseline PaO_2_/FiO_2_ ratio below 100, while 7 had a ratio between 100 and 199. The median ECMO duration was 15 days (IQR: 11–30). ECMO was discontinued due to clinical improvement in 17 patients, all of whom were later discharged from the ICU in stable condition, while 15 patients died. The incidence of hemolysis was significantly higher among patients on ECMO. Specifically, 81% (26 of 32) of patients undergoing ECMO exhibited hemolysis, whereas only 22% (4 of 18) of those not receiving ECMO were affected (*p* < 0.001). Furthermore, both markers of hemolysis, haptoglobin and hemopexin, were significantly lower in patients on ECMO compared to those without ECMO throughout the eight-day observation period (Figure 4). These findings highlight an association between ECMO treatment and an increased incidence of hemolysis, as evidenced by the reduced levels of the scavenger proteins haptoglobin and hemopexin.

#### 3.2.4. Incidence of Hemolysis in Patients Undergoing Continuous Renal Replacement Therapy

**Figure 5:**
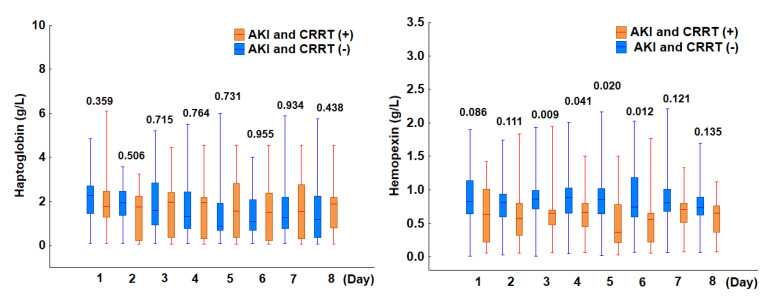
Comparison of haptoglobin (**left panel**) and hemopexin (**right panel**) levels between patients with and without AKI and CRRT. The box plots depict the median values (midline) and interquartile range (box) between the 25th and 75th percentiles, while the whiskers indicate the minimum and maximum values. For both parameters, the *p*-values shown for days 1–8 represent comparisons between patients with and without AKI and CRRT.

Continuous renal replacement therapy (CRRT), an extracorporeal life-support device, carries the potential risk of inducing hemolysis. In our analyzed cohort, 26% (*n* = 13) of patients were diagnosed with AKI stage 3 and required CRRT. Among them, CRRT was initiated within 24 h of ICU admission in seven patients, while the remaining six started treatment on days 2 or 3. Our analysis found no significant difference in the incidence of hemolysis between patients who received CRRT and those who did not. Specifically, hemolysis was observed in 69% (9 of 13) of CRRT patients, compared to 57% (21 of 37) in the non-CRRT group (*p* = 0.429). Although haptoglobin concentrations were similar between the two groups, hemopexin levels were significantly lower in CRRT patients, with statistically significant differences emerging between days 3 and 6 (Figure 5). Additionally, we investigated whether the concurrent use of extracorporeal membrane oxygenation (ECMO) and CRRT further increased the hemolysis risk. Our findings indicate that 14% of patients required both therapies, and all of them exhibited hemolysis (*p* = 0.020). These findings suggest the following: (1) while CRRT alone does not significantly increase the risk of hemolysis, its combination with ECMO markedly elevates its incidence; and (2) deficiency in hemopexin, a key scavenger of free heme, may contribute to the development of AKI and the subsequent need for CRRT in septic patients.

### 3.3. Prediction of ICU Mortality

Since hemolysis can influence the complex physiological response to infection and contribute to worse outcomes in patients with sepsis, we aimed to develop a predictive model for mortality in the analyzed cohort. In the present study, the ICU mortality was 54% (27 out of 50 patients). To assess the predictive value of haptoglobin and hemopexin concentrations alongside established biomarkers and clinical scores, both univariate and multivariate analyses were performed. Univariate analysis identified parameters such as haptoglobin level, age, clinical scores, and AKI stage 3 with the need for CRRT as significant predictors of mortality (Table 2). In multivariate analysis, a haptoglobin level below the reference range, advanced age, and AKI stage 3 remained independent predictors of mortality in this cohort.

## 4. Discussion

Haptoglobin and hemopexin serve as crucial scavenger proteins, binding free hemoglobin and free heme to mitigate their toxic effects and facilitate their clearance from circulation. Their depletion in plasma is a strong indicator of ongoing hemolysis, making them valuable tools for diagnosing and monitoring hemolytic conditions. In this observational study of septic patients with acute respiratory failure treated in the ICU, hemolysis was detected in 60% of the cohort. Notably, in some patients, reductions in plasma haptoglobin or hemopexin were evident as early as the day of ICU admission. The incidence of hemolysis was significantly higher in patients with septic shock and those receiving ECMO therapy. Logistic regression analysis revealed that low haptoglobin levels, along with advanced age and acute kidney failure stage 3, were significant predictors of mortality in this patient population.

Intravascular hemolysis may occur in sepsis, leading to elevated levels of extracellular hemoglobin and its porphyrin component, heme, which may exacerbate organ damage and increase the risk of death [2]. To counteract the toxic effects of free hemoglobin and heme, the body has developed protective mechanisms. Haptoglobin and hemopexin, by binding free hemoglobin and free heme, limit their toxic effects and facilitate their removal from the circulation [1]. Haptoglobin is an acute-phase protein that binds circulating cell-free hemoglobin dimers with high affinity. This binding prevents hemoglobin from entering tissues, thereby mitigating key harmful effects such as vascular injury and oxidative tissue damage [3]. These protective effects are especially important for the maintenance of kidney function, where heme-induced oxidative reactions lead to hemosiderin deposition, iron overload, lipid peroxidation, and tubular injury [16]. Notably, studies investigating the use of haptoglobin as a treatment for hemolysis have reported beneficial clinical effects without an increase in adverse events. Specifically, haptoglobin administration has been associated with a reduced incidence of acute kidney injury, likely by preventing hemosiderin deposition in renal tubular cells [17]. In sepsis-related hemolysis, hemopexin plays a similarly critical sequestering role by binding free heme, which is released from oxidized cell-free hemoglobin. As the primary scavenger of free heme, hemopexin complements haptoglobin’s role in neutralizing the toxic byproducts of hemolysis. By tightly binding heme, hemopexin prevents its participation in oxidative reactions with lipids, proteins, nucleic acids, and other biomolecules [2]. Although free heme readily translocates into endothelial cells, this process is effectively blocked once heme is bound to hemopexin. Consequently, heme sequestration by hemopexin provides extracellular protection against heme-driven oxidative damage, and inhibits heme-induced inflammation and the upregulation of adhesion molecules [18]. Heme is also an activator of toll-like receptor 4 (TLR4), and in endothelial cells, it promotes TLR4-mediated signaling that leads to increased expression of adhesion molecules and activation of NF-κB [19]. Hemopexin effectively blocks this heme-induced TLR4 activation. In addition, hemopexin attenuates the pro-inflammatory effects of heme, which can act synergistically with bacterial components such as lipopolysaccharides to amplify the inflammatory response. Liang et al. demonstrated that hemopexin-mediated sequestration of heme attenuates TLR4 and TLR2 agonist-induced cytokine production by macrophages, thereby limiting systemic inflammation in both the circulation and peripheral tissues [20]. Accumulating clinical evidence indicates that higher endogenous levels of haptoglobin and hemopexin are associated with reduced organ injury and improved outcomes in sepsis, likely due to the protective mechanisms described above. In a cohort study of septic ICU patients, Janz et al. reported that survivors had significantly higher plasma concentrations of both haptoglobin and hemopexin compared to non-survivors [7]. Similarly, Kubota et al., in a study of patients undergoing cardiac surgery using coronary artery bypass grafting (a setting characterized by substantial hemolysis), found that intraoperative hemopexin infusion was associated with a significantly lower incidence of postoperative acute kidney injury [21]. In sepsis, hemolysis may be exacerbated by extracorporeal circuits or blood transfusions. For example, veno-venous ECMO is known to elevate plasma levels of cell-free hemoglobin, which correlates with impaired pulmonary and renal function. A recent study by Bünger et al. in patients with ARDS receiving ECMO demonstrated that even moderate elevations in cell-free hemoglobin levels were significantly associated with increased mortality [22]. These findings suggest that adequate endogenous levels of haptoglobin and hemopexin may help to mitigate the deleterious effects of ongoing hemolysis in sepsis.

Haptoglobin not only plays a protective role by mitigating the toxic effects of cell-free hemoglobin, but also functions as an acute-phase protein that helps to trigger systemic inflammatory responses [23,24]. Experimental studies in haptoglobin-deficient mice have demonstrated significant impairments in immune responses to bacterial infections, including reduced B and T cell proliferation and abnormalities in their functional differentiation—effects that were partially reversed by administering recombinant haptoglobin [25]. Elevated plasma haptoglobin levels have been observed in sepsis. Verheij et al. reported significantly higher haptoglobin level in patients with sepsis measured at admission to ICU compared to healthy controls [8]. Similarly, Lan et al. demonstrated that ICU patients with septic shock had higher initial haptoglobin levels than patients without sepsis [6]. Our findings align with these studies, as half of the patients in our cohort exhibited elevated haptoglobin levels upon ICU admission, likely reflecting the systemic inflammatory response characteristic of sepsis.

Severe hypotension and hypoxia in septic shock can lead to tissue hypoperfusion, triggering metabolic stress and increasing RBC fragility. Multiple interconnected mechanisms contribute to hemolysis in septic shock, including mechanical RBC damage, oxidative stress, immune dysregulation, and microvascular perfusion disorders [3,26,27,28,29]. Animal studies have demonstrated that haptoglobin administration in septic shock exerts a protective effect by irreversibly neutralizing the toxicity of cell-free hemoglobin. This intervention has been shown to mitigate the adverse effects of hemolysis, significantly reducing shock severity and lowering mortality risk [30,31]. In our study, patients with septic shock at ICU admission had a significantly higher incidence of hemolysis than those without shock (86% vs. 24%). Moreover, both markers of hemolysis—haptoglobin and hemopexin—remained consistently lower in patients with septic shock throughout the observation period, reinforcing the strong association between septic shock and a higher incidence rate of hemolysis. Our findings align with those of Lan et al., who reported a significant decline in haptoglobin levels in patients with septic shock as disease severity increased, as reflected by higher SOFA scores [6]. However, our results contrast with those of Verheij et al., who found that while hemopexin levels were significantly lower in septic patients compared to healthy controls, indicating hemopexin consumption upon binding cell-free heme, haptoglobin levels were significantly higher in septic patients, likely reflecting the activation of systemic inflammation [8]. Notably, there was no difference in haptoglobin or hemopexin levels between sepsis and septic shock patients at ICU admission. The discrepancies between our and Verheij’s findings may be attributed to differences in measurement techniques. We analyzed haptoglobin and hemopexin levels using an immunonephelometric assay conducted in a hospital laboratory, a method specifically designed for in vitro diagnostics. Before incorporating haptoglobin and hemopexin measurements into routine clinical diagnostics, standardization of analytical methods should be established, prioritizing those validated for in vitro diagnostic use.

Our study cohort consisted of patients with acute respiratory failure, with ARDS confirmed in the majority. Emerging evidence suggests that the presence of red blood cells (RBCs) in the alveolar space may contribute to the pathophysiology of lung injury in ARDS and other pulmonary diseases by releasing cell-free hemoglobin into the alveolar space [32]. Both experimental and clinical studies have demonstrated that the alveolar epithelium is a primary target of cell-free hemoglobin during acute lung injury [33,34]. In a mouse model, intratracheal administration of cell-free hemoglobin induced acute lung injury, characterized by alveolar inflammation and disruption of the alveolar–capillary barrier [33]. Additionally, cell-free hemoglobin at concentrations detected in the alveolar spaces of ARDS patients was found to upregulate the expression of pro-inflammatory cytokines, including tumor necrosis factor-alpha, interleukin-6, interleukin-1 beta, and interleukin-12, in lung epithelial cells. This inflammatory cascade may further amplify inflammation, exacerbate lung injury, and contribute to worsening lung function.

The incidence rate of hemolysis in patients treated with ECMO varies depending on the patient population, ECMO type, and circuit characteristics [35,36,37]. Moreover, differences in defining hemolysis exist between studies, and various parameters have been used, such as plasma-free hemoglobin, lactate dehydrogenase, or haptoglobin and hemopexin. Intravascular hemolysis during ECMO is a side effect of blood circulating through extracorporeal systems and membrane oxygenators and subsequent mechanical damage to RBCs [38]. The significance of hemolysis in patients on ECMO has been increasingly recognized. Recently, Rezoagli et al. showed that haptoglobin levels were lower in those patients undergoing ECMO for ARDS who eventually died [9]. The deleterious effects of hemolysis may manifest in kidney function. Enhanced hemolysis has been reported during combined ECMO and CRRT compared with during ECMO alone [39,40]. Graw et al. demonstrated that in patients with ARDS, the occurrence of AKI at ECMO initiation increased with an increase in cell-free hemoglobin and a decrease in haptoglobin in a dose-dependent manner [41]. In our study, the incidence of hemolysis was significantly higher among patients on ECMO compared to those without ECMO (81% vs. 22%), with significant depletion in the markers haptoglobin and hemopexin throughout the whole study.

CRRT, as an extracorporeal therapy, carries a potential risk of inducing hemolysis. However, in our study, CRRT alone did not significantly increase the hemolysis risk. In contrast, the combination of CRRT with ECMO significantly elevated its incidence, with all patients receiving both therapies exhibiting hemolysis. Reports of hemolysis associated with CRRT in the literature are limited, suggesting that it is either a relatively minor issue or an under-recognized complication that warrants further investigation [42,43].

During severe hemolysis, the body’s protective mechanisms against cell-free hemoglobin toxicity become saturated, leading to glomerular filtration as the primary pathway for removing excess hemoglobin from the circulation. While renal clearance is a rapid and effective means of limiting hemoglobin exposure in other tissues, it can result in significant accumulation of cell-free hemoglobin in the kidneys, potentially causing renal damage [44]. Previous human and animal studies have demonstrated an association between severe intravascular hemolysis and acute kidney injury [41,45,46,47]. Findings from an animal study by Duel et al. indicate that hemolysis can cause kidney injury, with free heme accumulating in the renal cortex [45]. This accumulation triggers oxidative reactions that may lead to tubular damage and acute renal injury. The detrimental effects of hemolysis on kidney function have also been observed in human studies. For example, a study on patients undergoing on-pump cardiac surgery demonstrated a clear link between hemolysis and renal tubular injury [46,47]. Increased levels of circulating cell-free hemoglobin led to glomerular filtration of hemoglobin, causing tubular injury that further exacerbated renal damage. Moreover, peak plasma free hemoglobin levels were found to be a significant predictor of postoperative AKI. Importantly, plasma haptoglobin levels dropped significantly by the end of surgery and continued to decline postoperatively, suggesting active clearance of the haptoglobin–cell-free hemoglobin complex [47]. Similarly, in a study of critically ill patients with ARDS, decreased plasma haptoglobin and elevated cell-free hemoglobin levels were independently associated with a higher incidence of AKI [41]. These findings highlight the potential therapeutic benefit of exogenous haptoglobin supplementation, which should be further explored in clinical trials involving patients with severe ARDS. Our study confirmed that CRRT itself does not increase the incidence of hemolysis. However, we found that hemopexin deficiency may be linked to a higher risk of AKI and an increased need for CRRT in septic patients. Reduced hemopexin levels during hemolysis reflect increased consumption of this key scavenger protein as it binds and neutralizes free heme released from lysed RBCs. A decline in hemopexin suggests that the body’s capacity to clear free heme is overwhelmed, potentially exacerbating hemolysis-related complications, such as vascular damage, immune activation, and worsening renal dysfunction—all of which may contribute to the development of AKI.

Accumulating evidence suggests a role of hemolysis in sepsis and ARDS as a factor contributing to increased mortality [34,35]. The depletion in plasma hemopexin and haptoglobin indicates that the body’s protective mechanisms against cell-free hemoglobin toxicity become saturated, leading to cell-free hemoglobin and heme exposure in other tissues, potentially causing organ damage. The link between hemolysis and adverse outcomes has been previously established by other researchers. In a recent study by Rezoagly, low levels of haptoglobin accompanied by high carboxyhemoglobin levels were signs of an increased risk of incidence of secondary infections, such as ventilator-associated pneumonia, and higher ICU mortality in a population of ARDS patients on ECMO [9]. In another study conducted in a similar population of patients, hemolysis, assessed with measurements of haptoglobin and cell-free hemoglobin, was associated with significantly higher ICU mortality, and a haptoglobin plasma level of 0.39 g/L was identified as the cutoff value for mortality prediction [22]. In a study of septic shock patients, improved ICU mortality was linked to the concentration of haptoglobin in plasma; i.e., for patients with plasma haptoglobin <0.95 g/L, the mortality was 51%, while a higher concentration of haptoglobin was associated with a mortality of 34% [6]. In our study, a prediction model for mortality was developed, with a haptoglobin level below 0.30 g/L, advanced age, and a diagnosis of AKI stage 3 remaining independent predictors of mortality in the population of septic patients with acute respiratory failure. This finding indicates the importance of monitoring hemolysis for the prediction of patients’ outcomes.

Diagnosing sepsis-associated hemolysis requires integrating the clinical context with a panel of laboratory tests to identify RBC destruction and its systemic consequences. Sensitivity and specificity are crucial measures of a diagnostic test’s accuracy for identifying patients with intravascular hemolysis. Sensitivity, in the context of hemolysis, refers to a test’s ability to correctly identify individuals with intravascular hemolysis, while specificity refers to the ability of a test to correctly identify individuals without hemolysis. Tests for hemolysis fall into two broad categories: direct tests, which detect components released from lysed RBCs or structural changes in the cells; and indirect tests, which assess the biochemical effects of RBC destruction. Examples of direct tests include measurement of plasma free hemoglobin, examination of a peripheral blood smear, and the direct antiglobulin test, while examples of indirect tests include measurement of haptoglobin, hemopexin, and lactate dehydrogenase (LDH). Among the direct tests, plasma free hemoglobin is a commonly used laboratory marker for detecting intravascular hemolysis [22,48]. Although it is sensitive to RBC rupture within the circulation, its specificity is limited by factors such as improper sample collection or handling, which can cause artificial elevations and false-positive results. Moreover, the reproducibility of this test is a concern. For example, Adamzik et al. compared four different methods (one immunosorbent assay and three spectrophotometric assays) to determine plasma free hemoglobin concentrations in septic patients, and found substantial discrepancies in the median values among the methods [49]. These differences highlight the issue of inter-laboratory variability, which complicates the comparison of extracellular hemoglobin results across studies. Due to these technical limitations, plasma cell-free hemoglobin assays are not available as part of routine testing in our hospital laboratory. Other direct tests can also aid in specific clinical scenarios. For instance, a blood smear can help to detect morphological abnormalities and fragmented RBCs, which may indicate microangiopathic hemolysis. Similarly, the direct antiglobulin test is useful in evaluating suspected autoimmune hemolytic anemia in septic patients. Among indirect tests for intravascular hemolysis, measurements of haptoglobin and/or hemopexin have been often used. Haptoglobin is sensitive to intravascular hemolysis, as it binds free hemoglobin released from lysed RBCs. Thus, low haptoglobin is often an early marker of hemolysis. However, its specificity is limited. Haptoglobin is an acute-phase reactant and may be elevated in inflammation, infection, or tissue injury, which are common in sepsis [23,24]. This can mask the expected decrease in hemolysis, making interpretation challenging in septic patients. Haptoglobin measurement is generally reproducible, with CE IVD-certified standardized assays available in most hospital laboratories for detecting hemolysis in patient samples. Hemopexin, similarly to haptoglobin, is sensitive to intravascular hemolysis, as it binds free heme, which is released after hemoglobin degradation. Plasma concentration of hemopexin decreases during significant hemolysis in cases severe red-cell destruction. Moreover, hemopexin is specific to hemolysis because it is not an acute-phase protein, and therefore is less influenced by inflammation. This makes it a valuable adjunct marker of hemolysis in complex clinical contexts like sepsis. Haptoglobin measurement is generally reproducible, with standardized assays available in hospital laboratories. Lactate dehydrogenase (LDH) is a commonly used laboratory parameter in the assessment of intravascular hemolysis. LDH is an intracellular enzyme that catalyzes the interconversion of lactate and pyruvate during anaerobic glycolysis. It is sensitive to intravascular hemolysis, as RBCs contain high concentrations of LDH, which is released into the plasma upon RBC rupture. However, LDH has low specificity for hemolysis because it is an ubiquitous enzyme found in multiple tissues, including the liver, heart, kidneys, muscles, and lungs. Consequently, elevated LDH levels may reflect a broad range of conditions, such as tissue injury, organ dysfunction, or inflammation [50,51]. Brauckmann et al. demonstrated that bacterial lipopolysaccharide can directly interact with RBC membranes, causing membrane disruption and hemolysis, which are accompanied by increased LDH activity [52]. In the setting of sepsis, LDH measurement provides insight into the extent of cellular injury and organ dysfunction, rather than serving solely as a hemolysis marker. Elevated LDH levels in sepsis may arise from multiple, overlapping mechanisms, including the following: tissue hypoxia and necrosis due to impaired perfusion and oxygen delivery; inflammatory cell damage resulting from the systemic inflammatory response; organ dysfunction; and hemolysis. These processes are integral to the host response to infection and systemic inflammation [53]. LDH elevation in sepsis has been associated with greater disease severity and worse clinical outcomes. Notably, increased LDH levels have also been linked to respiratory dysfunction and identified as a predictor of respiratory failure in COVID-19 patients [54,55]. In our study, LDH activity was elevated in patients both with and without laboratory evidence of hemolysis, with no statistically significant difference between the groups. This finding suggests that in septic patients, LDH activity more accurately reflects the overall burden of cellular injury and organ dysfunction, rather than serving as a specific marker of hemolysis. Finally, the reproducibility of LDH measurement is high when performed under standardized conditions; it is a routine, widely available laboratory test.

This study has several limitations. First, we assessed hemolysis indirectly, as direct measurements, such as plasma cell-free hemoglobin assays, are no longer available as part of routine testing in our hospital laboratory. In a study by Adamzik et al., four different methods for measuring cell-free hemoglobin were evaluated, revealing significant variability, with measured values differing by up to 100% [49]. Additionally, discrepancies in test results may be attributed to factors such as sample handling, storage, and processing, which may introduce ex vivo hemolysis during blood collection or processing, leading to inaccurate results. Due to these technical challenges and a lack of standardization, extracellular hemoglobin measurements may be difficult to compare across studies. In contrast, measurements of plasma haptoglobin and hemopexin, both of which are widely available in hospital laboratories, provide a practical and reliable approach for detecting and monitoring hemolysis in routine clinical practice. A second limitation is the selected study population of septic patients with acute respiratory failure. For generalization of the results of the incidence rate of hemolysis in sepsis and the impact of hemolysis on patients’ outcomes, further studies should be conducted on a general population of septic patients. Third, the retrospective nature of the study presents inherent limitations, including dependence on existing records rather than prospective measurements. Therefore, future prospective studies are needed to provide more robust and conclusive evidence. Such studies should aim to include diverse patient populations to enhance generalizability. In addition, prospective designs would allow for standardized, dynamic monitoring of hemolysis biomarkers, facilitate the evaluation of treatment effects, and support time-resolved analyses of the association between hemolysis and clinical outcomes. Ultimately, these efforts could deepen our understanding of the causal role of hemolysis in sepsis-related organ dysfunction, and aid in identifying potential therapeutic targets. One such target is haptoglobin supplementation in patients with significantly reduced plasma levels. Emerging evidence suggests that haptoglobin supplementation may help to prevent or manage complications related to hemolysis in various clinical settings [17]. For example, in cardiac surgery patients, intraoperative administration of haptoglobin was independently associated with a lower risk of postoperative acute kidney injury [21]. Investigating the use of exogenous haptoglobin in septic patients with evidence of hemolysis may offer a novel therapeutic approach to reduce hemoglobin-mediated toxicity, improve clinical outcomes, and restore hemoglobin-scavenging capacity in critically ill patients.

## 5. Conclusions

Our study underscores the critical role of hemolysis in sepsis and acute respiratory failure, highlighting its strong association with organ dysfunction. The incidence of hemolysis is notably high, particularly among patients with septic shock and those requiring extracorporeal therapies. Hemolysis significantly contributes to renal impairment, with hemopexin deficiency correlating with an increased incidence of acute kidney injury (AKI) and the necessity for continuous renal replacement therapy (CRRT). The depletion of plasma haptoglobin and hemopexin serves as a reliable indicator of ongoing hemolysis, with low haptoglobin levels, in particular, emerging as significant predictors of mortality. These findings underscore the importance of monitoring hemolysis in septic patients, especially given that measuring plasma haptoglobin and hemopexin concentrations is feasible in most hospital laboratories, rendering them practical and accessible biomarkers for the detection and monitoring of hemolysis in routine clinical practice. Given the protective role of haptoglobin in neutralizing cell-free hemoglobin toxicity, future research should explore its therapeutic potential in mitigating hemolysis-related complications in sepsis.

## Figures and Tables

**Table 1:** Baseline characteristics of study population.

Variable	All	Group 1	Group 2	*p*-Value
	*n* = 50	*n* = 30	*n* = 20	
**Age (years)**	47 (38–59)	46 (38–57)	50 (38–62)	0.469
**Gender, male *n* (%)**	33 (66)	23 (70)	10 (30)	0.050
**APACHE II score**	14 (11–19)	15 (12–21)	14 (11–18)	0.481
**SOFA score**	9 (8–11)	9 (8–12)	8 (7–10)	0.036
**Comorbidities, *n* (%):**				
Hypertension	10 (20)	6 (60)	4 (40)	0.645
Coronary heart disease	6 (12)	3 (50)	3 (50)	0.455
Diabetes	6 (12)	4 (67)	3 (33)	0.544
Obesity	14 (28)	10 (71)	4 (29)	0.241
Malignancy	2 (4)	1 (50)	1 (50)	0.644
**Laboratory parameters:**				
Leukocytes, 10^3^/uL	15 (11–20)	17 (11–24)	14 (10–18)	0.238
Hemoglobin, (g/dL)	11.8 (10.4–12.8)	11.6 (10.4–12.2)	12.0 (10.2–13.7)	0.519
Platelets, (10^9^/L)	233 (173–302)	204 (170–274)	271 (201–304)	0.246
Fibrynogen, (g/L)	4.5 (3.1–6.7)	4.8 (2.6–6.9)	4.5 (3.9–6.1)	0.613
D-dimers, (mg/L)	5.1 (1.9–17.6)	5.1 (1.9–17.6)	5.0 (2.0–18.7)	0.858
ATIII, (%)	80 (67–95)	79 (65–93)	83 (67–104)	0.319
CRP, mg/L	115 (61–218)	112 (37–199)	126 (66–254)	0.227
PCT, (ng/mL)	0.37 (0.15–1.07)	0.5 (0.16–2.00)	0.31 (0.14–0.88)	0.254
LDH, (U/L)	664 (498–889)	610 (479–865)	747 (553–1030)	0.258
**Blood transfusion, *n* (%)**	29 (58)	19 (63)	10 (50)	0.349
**ICU length of stay, (day)**	23 (12–39)	22 (12–39)	28 (15–41)	0.613
**Hospital length of stay, (day)**	29 (16–51)	25 (12–51)	32 (18–50)	0.451
**ICU mortality, *n* (%)**	27 (54)	18 (60)	9 (45)	0.297
**Hospital mortality, *n* (%)**	29 (58)	19 (63)	10 (50)	0.349

APACHE II, Acute Physiology and Chronic Health Evaluation II; SOFA, Sequential Organ Failure Assessment; AT III, antithrombin III; CRP, c-reactive protein; PCT, procalcitonin; LDH, lactate dehydrogenase; ICU, intensive care unit. Continuous variables are presented as medians (25th–75th percentiles), categorical variables are summarized as counts and fractions. *p*-value represents differences between groups.

**Table 2:** Univariable and multivariable logistic regression analysis for ICU mortality.

	Univariable			Multivariable		
Variables	OR	95%CI	*p*-Value	OR	95%CI	*p*-Value
Age	1.1	1.0–1.2	<0.001	1.2	1.01–1.3	0.002
Gender	1.5	0.5–4.9	0.481			
Septic shock	2.3	0.7–7.7	0.198			
APACHEII	1.2	1.1–1.4	0.004			
SOFA	1.4	1.1–1.9	0.018			
ECMO	0.4	0.1–1.5	0.181			
AKI stage 3	17.6	2.1–150.0	0.008	22.2	1.4–358.1	0.029
Haptoglobin < 0.3 mg/L	4.6	1.1–19.3	0.037	27.1	2.7–276.9	0.005
Hemopexin < 0.5 mg/L	1.9	0.6–5.8	0.267			
Hb	1.1	0.8–1.5	0.562			
LDH	1.1	1.0–1.0	0.189			
Secondary infection	0.7	0.2–2.1	0.487			
Hypertension	2.3	0.5–10.3	0.246			
Diabetes	5.0	0.5–46.4	0.156			
Obesity	1.8	0.5–6.4	0.365			
Coronary artery disease	5.0	0.5–46.4	0.157			

## Data Availability

The data presented in the study are available on request from the corresponding author. The data have not been made publicly available because they contain information that could compromise the privacy of the study participants.
